# A novel *NR5A1* variant in an infant with elevated testosterone from an Australasian cohort of 46,XY patients with disorders of sex development

**DOI:** 10.1111/cen.12012

**Published:** 2013-03-12

**Authors:** Joyce Y Wu, Ivan N McGown, Lin Lin, John C Achermann, Mark Harris, David M Cowley, Salim Aftimos, Kristen A Neville, Catherine S Choong, Andrew M Cotterill

**Affiliations:** * Department of Clinical Chemistry, Mater HospitalSouth Brisbane, QLD, Australia; † Developmental Endocrinology Research Group, Clinical and Molecular Genetics Unit, University College London Institute of Child Health, University College LondonLondon, UK; ‡ Department of Paediatric Endocrinology, Mater Children's HospitalSouth Brisbane, QLD, Australia; § Northern Regional Genetics Service, Auckland HospitalAuckland, New Zealand; ¶ Department of Endocrinology, Sydney Children's HospitalRandwick, NSW, Australia; ** Department of Endocrinology and Diabetes, Princess Margaret Hospital for Children, School of Paediatrics and Child HealthUniversity of Western Australia Subiaco, WA, Australia; †† University of QueenslandBrisbane, QLD, Australia; ‡‡ Mater Medical Research InstituteSouth Brisbane, QLD, Australia

## Abstract

**Background:**

*NR5A1* loss-of-function mutations are increasingly found to be the cause of 46,XY disorders of sex development (DSD).

**Objective:**

To determine the presence of *NR5A1* mutations in an Australasian cohort of 17 46,XY DSD patients with presumed androgen insensitivity syndrome (AIS) who were negative for androgen receptor gene (*AR*) mutation.

**Design:**

Exons 2-7 of *NR5A1* were PCR amplified and sequenced. Gene expression and cellular localization studies were performed on a novel *NR5A1* variant c.74A>G (p.Y25C) identified in this study.

**Results:**

We identified one novel mutation, c.74A>G (p.Y25C) in a patient characterized by penoscrotal hypospadias with bifid scrotum. He had elevated testosterone and gonadotropins in early infancy. Functional analysis of p.Y25C *in vitro* demonstrated reduced transcriptional activation by SF-1 and partially impaired nuclear localization in a proportion of transfected human adrenal NCI-H295R cells.

**Conclusion:**

This is the first reported case of a DSD patient with a *NR5A1* mutation and elevated testosterone levels. Our finding supports evaluation of *NR5A1* mutations in 46,XY DSD patients with a range of testosterone levels.

## Introduction

Disorders of sex development (DSD) encompass all the congenital conditions in which development of chromosomal, gonadal or anatomic sex is atypical. One in 4500 births requires genetic and endocrine studies because of abnormalities of their external genitalia. Definitive diagnosis is made in only 50% of 46,XY children with DSD.^1^

Recently, several investigators have reported heterozygous loss-of-function mutations in the nuclear receptor subfamily five group A member 1 gene (*NR5A1*) in patients with clinical features of androgen insensitivity syndrome (AIS) but without androgen receptor gene (*AR*) mutations.^2^ Mutations in *AR*, which is located on chromosome Xq11-q12, is responsible for AIS. The detection rate for *AR* mutations in Complete AIS (CAIS) range from 66·7% to 83%, whereas for Partial AIS (PAIS) patients, the detection rate for *AR* mutations range from 13·6% to 28%.^3^,^4^ AIS is characterized by a clinical spectrum ranging from phenotypically female patients (CAIS) to decreased virilization (PAIS) in 46,XY individuals with normal or elevated androgen levels.^5^

The *NR5A1* gene is mapped to 9q33 and consists of seven exons spanning approximately 30 kb. Exon 1 is untranslated. It encodes Steroidogenic Factor-1 (SF-1) also known as Adrenal 4-Binding Protein (Ad4BP). SF-1 is a 461 amino acid protein belonging to the NR5A subfamily of nuclear receptors. It is a transcription factor that binds to specific DNA sequences in target genes and regulates transcription.^6^ The target genes are expressed throughout the hypothalamic-pituitary–adrenal/gonadal axis and include the steroid hydroxylase genes, luteinizing hormone receptor, adrenocorticotrophin receptor, StAR protein and SOX9.^6^,^7^ Thus, SF-1 has a central role in regulating adrenal development, gonadal determination and differentiation and in the hypothalamic-pituitary control of reproduction and metabolism.^6^

In XY mice with homozygous *NR5A1* deletions, there is impaired adrenal development, complete testicular dysgenesis with Müllerian structures and female external genitalia. Two human patients with these clinical features and *NR5A1* mutations have been reported.^8^,^9^ Varying degrees of functional impairment in SF-1 can be associated with a wide range of reproductive phenotypes without adrenal involvement, extending from ambiguous genitalia, hypospadias to male infertility.^2^,^10^–^13^

We evaluated the presence of *NR5A1* mutations in an Australasian cohort of 17 46,XY DSD patients with presumed AIS who were negative for *AR* mutations.

## Materials and methods

### Patients

*NR5A1* testing was performed in a cohort of 17 patients who had presumed androgen insensitivity syndrome but who were negative for *AR* mutations. Six of these patients have been previously reported.^3^ Of the 17 patients, 15 were diagnosed with PAIS and two with CAIS.

### Molecular analysis of *NR5A1*

Molecular testing was performed at the National Association of Testing Authorities (NATA) accredited Molecular Laboratory, Mater Pathology, Mater Health Services, Brisbane, QLD.

Oligonucleotide primers were designed with Primer 3 software (http://frodo.wi.mit.edu/primer3/primer3_code.html) and checked for the presence of SNPs using the SNPCheck2 program (https://ngrl.manchester.ac.uk/SNPCheckV2/snpcheck.htm). Primer sequences are available on request.

Genomic DNA was extracted from peripheral blood leucocytes, and exons 2-7 of the *NR5A1* gene were amplified using a GC-Rich PCR system (Roche/Boehringer Mannheim, Mannheim, Germany). PCR products were then purified with the High Pure PCR Product Purification Kit (Roche/Boehringer Mannheim) and sequenced using the Big Dye Terminator V3·1 Cycle Sequencing Kit (Applied Biosystems, Scoresby, vic., Australia). Sequences were read on an ABI 3130xl Genetic Analyser (Applied Biosystems) at the Griffith University Sequencing Facility (Griffith University, Nathan, QLD, Australia). All sequences were compared with consensus sequences for *NR5A1* genomic and mRNA sequences. DNA mutation numbering is based on GenBank reference DNA sequence NM_004959·4, with the A of the ATG initiation codon designated +1 (http://www.hgvs.org/mutnomen).

### Transient gene expression assays

An expression vector containing the p.Y25C change was generated by site-directed mutagenesis (QuikChange, Stratagene, Amsterdam, the Netherlands) using wild-type (WT) pCMXSF-1 as a template. Transient transfection studies were performed in 96-well plates using tsa201 human embryonic kidney cells. These cells do not express SF-1 and have been used widely in studies of SF-1 function in the past.^9^,^11^,^12^ Empty (−), WT or mutant SF-1 expression vectors (2 ng/well; p.G35E, p.Y25C) were co-transfected with the SF-1-responsive minimal promoter of *Cyp11a* linked to luciferase (100 ng/well) using lipofectamine 2000 (Invitrogen, Paisley, UK). Cells were lysed 24 h later and assayed for luciferase activity (Dual Luciferase Reporter Assay system, Promega; FLUOstar Optima, BMG Labtech, Aylesbury, UK), with standardization for *Renilla* co-expression.

### Studies of cellular localization

A p.Y25C mutant GFP-SF-1 construct was generated by site-directed mutagenesis in a pAcGFP-C1 vector (Clontech, Oxford, UK) to produce a mutant fusion protein with a GFP tag at the amino-terminus of SF-1. Empty (−), WT and mutant p.Y25C pAcGFP-C1SF-1 expression vectors (0·8 μg) were transfected into SF-1 expressing NCI-H295R human adrenal cells. After 24 h, cells were fixed and counterstained with Vectashield containing DAPI (Vector Laboratories, Peterborough, UK). Images were taken on a confocal microscope.

### Reproductive hormone assays

Testosterone was analysed on the Spectria Testosterone RIA coated tube (Orion Diagnostica, Finland). Dihydrotestosterone was assayed using an in-house RIA after extraction and partition chromatography. LH and FSH were analysed on Immulite 2000 (Bio-Mediq DPC Pty Ltd, Los Angeles, CA, USA).

## Results

### Mutation analysis of patients

Molecular analysis revealed four patients to be heterozygous for a c.437G>C (p.G146A) sequence variant and one patient to be heterozygous for a c.74A>G (p.Y25C) sequence variant.

The c.437G>C sequence variant is a previously described variant located in the hinge region.^7^,^14^ Some consider it a polymorphism as previous p.G146A *in vitro* functional studies have demonstrated unaltered SF-1 transactivation activity.^15^ Therefore, these patients will not be further discussed in this study.

**Fig. 1:**
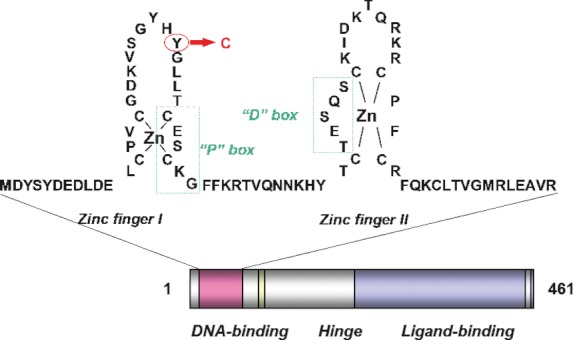
The c.74A>G alteration in this patient causes a tyrosine to cysteine change at codon 25 (p.Y25C). This amino acid is highly conserved and affects a codon located within the first zinc finger DNA-binding domain (DBD).

The c.74A>G is a novel variant that has not been found in previous studies of controls.^13^ This change results in an amino acid change from a highly conserved tyrosine to cysteine at codon 25 (p.Y25C). This amino acid is situated in the first zinc finger DNA-binding domain (See Fig. 1). This appears to be a *de novo* change as both parents are phenotypically normal and were found to be negative for this variant.

The proband was the first child for the couple. He was born at 41 + 1 week following an uneventful pregnancy. At birth, he had penoscrotal hypospadias with a small phallus (20 mm long), chordee, a ventrally deficient prepuce, bifid scrotum and two scrotal testes. His pelvic and renal ultrasound demonstrated 0·5–1 ml testes bilaterally in the scrotum with normal kidneys bilaterally and no Müllerian structures. Hormone results shortly after birth showed high testosterone levels (See Table 1).

The provisional diagnosis was PAIS in this patient. There was no family history of DSD and/or adrenal insufficiency. At age 4, he had no evidence of adrenal insufficiency on formal short synacthen testing.

**Table 1:** Hormone results at two periods shortly after birth

	13 days old	1 month + 18 days old
Testosterone (nm)	10·8↑ (RI 3·1–10)^2^	25 ↑(RI 1·8–16·6)^2^
Dihydrotestosterone (nm)	4·2↑ (RI 0·17–2·1)^16^	5·3 ↑ (RI 0·4–2·9)^16^
LH (IU/l)	7·9↑ (RI 2·7–7·8)^2^	1·2 (RI 0·12–4·8)^2^
FSH (IU/l)	3·9↑ (RI 1·7–3·5)^2^	1·6 (RI 0·6–5·5)^2^

RI, Reference Interval.

### Functional analyses

**Fig. 2:**
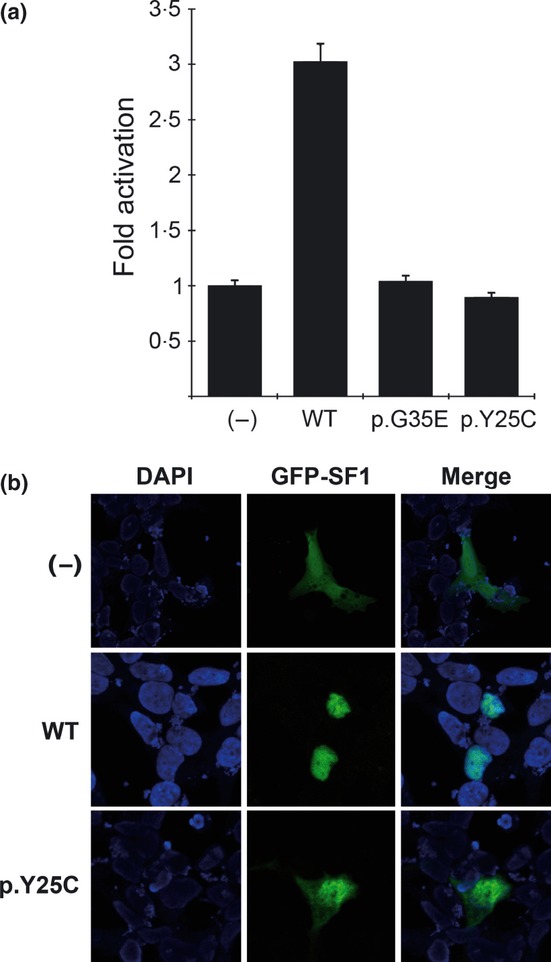
(a) Assays showing activation of a *Cyp11a1* promoter by wild-type (WT) SF-1 and impaired transcriptional activity by the p.Y25C mutant. Relative luciferase activity was measured and expressed as fold activation above baseline (empty vector). Results are shown as the mean ± sem of three independent experiments, each performed in triplicate. (−), empty vector; WT, wild-type; p.G35E, a known loss-of-function mutation. (b) Immunocytochemistry to show cellular localization of SF-1 linked to GFP. Wild-type (WT) SF-1 shows strong nuclear localization, whereas the p.Y25C mutant GFP-SF-1 construct shows partially impaired nuclear localization in a proportion of cells.

The p.Y25C variant of *NR5A1* showed impaired transcriptional activation of the *Cyp11a* promoter (Fig. 2a). In a substantial proportion of cells, p.Y25C mutant SF-1 showed partially impaired nuclear localization (Fig. 2b).

## Discussion

Currently, over 50 different *NR5A1* mutations have been reported in humans.^15^,^17^,^18^ These mutations include missense mutations, nonsense mutations caused by nucleotide deletions and duplications, a 3 Mb deletion, a 970 kb deletion and a deletion in exon 2 and 3. The degree of gonadal dysgenesis and underandrogenization varies from mild to severe, with three mutations also causing adrenal failure. All cases of 46,XY DSD (except for two homozygous mutations) are heterozygous for the mutation, indicating that SF-1 dosage is critical.^8^,^9^ The varying locations of the different *NR5A1* mutations suggest the absence of a mutation hot spot and the lack of a founder effect.

In our study of 17 patients with presumed androgen insensitivity but with no *AR* mutation, one patient was found to have a *de novo* mutation in *NR5A1* which caused reduced SF-1 transcriptional activation with partially impaired nuclear localization in a significant proportion of transfected cells. To our knowledge, this is the first patient described with a *NR5A1* loss-of-function mutation and elevated testosterone shortly after birth. It is known that within 24 h after birth and then at 1–2 months of life, there are physiologically high levels of testosterone that can be as informative as the hCG stimulation test.^19^ Despite one of our patient's levels being taken outside this period, his level was still high. Although *NR5A1* mutations are known to cause dysgenetic testes and/or impaired androgen synthesis, there have been previously published cases of affected 46, XY infants with normal testosterone levels.^2^,^20^ In the first described case, the baby had a perineal urogenital sinus, a genital tubercle measuring 12 mm in length, palpable gonads (12 × 5 mm) in the bilateral inguinal regions and a hypotropic scrotum or corrugation of major labia.^2^ The phenotype in the second case described may be due to other genes in addition to *NR5A1* contained in the deletion of chromosome 9q33.3 identified in this child. The phenotypically female infant was noted to have clitoromegaly at 10 weeks of age. She was then found to have a shallow vaginal entrance with a 1 ml gonad palpable in the left labium. Abdominal ultrasonography showed no uterus or Müllerian structures.^20^ Recently, two more *NR5A1* cases with normal testosterone at infancy were published in a cohort study. One patient had scrotal hypospadias, bilateral cryptorchidism and no Müllerian structures. Another had scrotal hypospadias, intraabdominal testes and presence of Müllerian duct remnants.^15^ Our patient's phenotype suggests that there was a sufficient amount of anti-Müllerian hormone (AMH/MIS) for Müllerian regression and testosterone for Wolffian development. There must have been low dihydrotestosterone or dihydrotestosterone insensitivity, however, at least during the critical period of external genitalia formation (between 8 and 14 weeks gestation).^12^,^21^ Elevated testosterone in this patient is unexpected; however, the mechanisms underlying the variable effect of *NR5A1* mutations are still not clearly understood. This is illustrated by the previously mentioned study of a baby with normal testosterone levels whose sibling (a 46, XY female) had the same mutation but was less virilized and had low testosterone production.^2^ It therefore appears that differences in phenotypes and Leydig cell function exist even within a family with the same mutation and other genetic or environmental factors may affect testosterone production.^2^ It may be that in some cases disruption of SF-1 is associated with functional androgen resistance^22^ or altered Leydig cell maturation and hyper-responsiveness to postnatal LH stimulation. These possible explanations for the relatively high androgen levels found here are speculative, but it will be of great interest to know whether *NR5A1* changes can be found in further systematic studies of children with high or normal androgen concentrations who have been diagnosed as having *AR* negative PAIS.

This case illustrates that mutations of *NR5A1* may be an important genetic cause in 46, XY DSD patients with a range of testosterone levels, and all patients negative for *AR* mutations should be considered for *NR5A1* gene analysis even if testosterone biosynthesis is elevated. It will be of interest to note whether the capacity to produce testosterone is maintained to enable normal progression of puberty and development of secondary sexual characteristics. Initial evidence suggested that with increasing age, there will be decreasing testosterone as one study found that 4% of otherwise healthy men with unexplained azoospermia carried mutations in *NR5A1* and that these men may also be at risk of endocrine dysfunction with failing testosterone with increasing age.^13^ However, recently, two studies presented in total four 46,XY patients with mutations in *NR5A1* who entered puberty spontaneously with normal testosterone levels.^18^,^23^ It will be interesting to document future testosterone levels in these patients to elucidate whether there is premature failure of testosterone production and to determine their fertility potential.

Both parents were found to be negative for the mutation found in their son. *De novo* mutations have been documented in the majority of reported *NR5A1* cases where the mode of inheritance is known.^24^ Interestingly, around 30% are documented to be inherited from the mother in a sex-limited dominant fashion, in that mothers may carry the mutation without ovarian dysfunction and pass it onto sons who are affected (mimicking X-linked inheritance).^17^ For the remainder, the inheritance is unknown, autosomal recessive or from their phenotypically normal fathers in rare instances.^15^,^24^ Thus, the absence of a family history should not detract from the possibility of *NR5A1* mutations.

The finding of an *NR5A1* mutation is important in the management of an infant with 46,XY DSD, as this diagnosis has implications for genetic counselling and future treatment. Unlike AIS, children with *NR5A1* mutations should theoretically respond to normal levels of exogenous testosterone. It is unclear whether 46,XY DSD patients with *NR5A1* mutations with the presence of testes and raised as males will be at risk of gonadal malignancy; however, it is likely that they will have progressive testicular changes over time with implications for fertility.^11^,^13^ This knowledge will influence sex assignment and family planning. As patients can have adrenal insufficiency, this knowledge will prepare patients and allow health professionals to initiate closer evaluation. Mutation carrier mothers and sisters of 46,XY DSD patients may also be at risk of premature ovarian insufficiency and possibly adrenal insufficiency.^25^ Females might also appear to be asymptomatic but pass on the mutation with the potential of having 46,XY DSD children. Being aware of these possibilities will help with genetic counselling and prepare the families and allow closer follow-up.

For the remainder of 46,XY DSD patients still without a molecular diagnosis other genes should be investigated. Evidence suggests that molecular analysis of *SRD5A2* for 5α-reductase deficiency and *HSD17B3* for 17β-hydroxysteroid dehydrogenase-3 deficiency are important as endocrine testing are potentially unreliable.^26^,^27^ This might also be the case with Leydig cell hypoplasia caused by inactivating mutation of the LH receptor.^28^ With the rapid advancements in genome sequencing, it is likely that simultaneous testing for all these genes will become more common in future.

In summary, we identified one novel *NR5A1* mutation in an Australasian cohort of 17 46,XY DSD patients who had previously been assessed for *AR* mutations. As *AR* mutations are only detected in 13·6–28% of patients with clinical diagnosis of PAIS, it is important for other diagnoses, including *NR5A1* mutations, to be considered.^3^,^4^ The high testosterone measurement in this case highlights the biochemical variability associated with SF-1/*NR5A1* mutations.
